# Evaluation of leucine-rich alpha-2 glycoprotein as a biomarker of fetal infection

**DOI:** 10.1371/journal.pone.0242076

**Published:** 2020-11-19

**Authors:** Etsuko Kajimoto, Masayuki Endo, Minoru Fujimoto, Shinya Matsuzaki, Makoto Fujii, Kazunobu Yagi, Aiko Kakigano, Kazuya Mimura, Takuji Tomimatsu, Satoshi Serada, Makoto Takeuchi, Kiyoshi Yoshino, Yutaka Ueda, Tadashi Kimura, Tetsuji Naka

**Affiliations:** 1 Department of Obstetrics and Gynecology, Osaka University Graduate School of Medicine, Osaka, Japan; 2 Department of Obstetrics and Gynecology, Japan Community Health Care Organization Osaka Hospital, Osaka, Japan; 3 Department of Children and Women’s Health, Osaka University Graduate School of Medicine, Osaka, Japan; 4 Division of Health Science, Graduate School of medicine, StemRIM Institute of Regeneration-Inducting Medicine, Osaka University, Osaka, Japan; 5 Center for Intractable Immune Disease, Kochi Medical School, Kochi University, Kochi, Japan; 6 Department of Pathology, Osaka Women’s and Children’s Hospital, Osaka, Japan; 7 Department of Obstetrics and Gynecology, School of Medicine, University of Occupational and Environmental Health, Kitakyushu, Japan; 8 Laboratory of Immune Signal, National Institutes of Biomedical Innovation, Health and Nutrition, Osaka, Japan; University of Insubria, ITALY

## Abstract

This study aimed to determine the association between umbilical cord leucine-rich alpha-2 glycoprotein (LRG) and fetal infection and investigate the underlying mechanism of LRG elevation in fetuses. We retrospectively reviewed the medical records of patients who delivered at Osaka University Hospital between 2012 and 2017 and selected those with histologically confirmed chorioamnionitis (CAM), which is a common pregnancy complication that may cause neonatal infection. The participants were divided into two groups: CAM with fetal infection (CAM-f[+] group, *n =* 14) and CAM without fetal infection (CAM-f[−] group, *n =* 31). Fetal infection was defined by the histological evidence of funisitis. We also selected 50 cases without clinical signs of CAM to serve as the control. LRG concentrations in sera obtained from the umbilical cord were unaffected by gestational age at delivery, neonatal birth weight, nor the presence of noninfectious obstetric complications (all, *p* > 0.05). Meanwhile, the LRG levels (median, Interquartile range [IQR]) were significantly higher in the CAM-f(+) group (10.37 [5.21–13.7] μg/ml) than in the CAM-f(−) (3.61 [2.71–4.65] μg/ml) or control group (3.39 [2.81–3.93] μg/ml; *p* < 0.01). The area under the receiver operating characteristic (ROC) curve of LRG for recognizing fetal infection was 0.92 (optimal cutoff, 5.08 μg/ml; sensitivity, 86%; specificity, 88%). In a mouse CAM model established by lipopolysaccharide administration, the fetal LRG protein in sera and *LRG* mRNA in the liver were significantly higher than those in phosphate-buffered saline (PBS)-administered control mice (*p* < 0.01). *In vitro* experiments using a fetal liver-derived cell line (WRL68) showed that the expression of *LRG* mRNA was significantly increased after interleukin (IL)-6, IL-1β, and tumor necrosis factor- alpha (TNF-α) stimulation (*p* < 0.01); the induction was considerably stronger following IL-6 and TNF-α stimulation (*p* < 0.01). In conclusion, LRG is an effective biomarker of fetal infection, and fetal hepatocytes stimulated with inflammatory cytokines may be the primary source of LRG production *in utero*.

## Introduction

Recently, neonatal morbidity rates have declined, owing to the availability of pregnancy screening tests and antibiotic treatment for mothers and neonates. However, fetal infections remain a substantial clinical problem and the cause of neonatal morbidity and mortality [1, 2]. Some fetal infections progress to early-onset neonatal sepsis, which occurs in approximately 0.77 to 1 per 1000 live births in the United States [3–5]. In terms of long-term outcomes, fetal infection is a risk factor for chronic lung disease and cerebral palsy [1, 6, 7]. One of the major etiologies of fetal infection is chorioamnionitis (CAM) [8, 9]. Hence, fetal infection in mothers with CAM must be identified to initiate intensive treatment immediately and decrease the risk of neonatal morbidity.

Infection is typically accompanied by increased levels of serum proteins known as acute-phase reactants (APRs) [10]. Changes in APR levels are not specific to infection, but such abnormalities generally indicate the presence and intensity of inflammatory conditions, including infection. Among the APRs, C-reactive protein (CRP) is the most commonly used biomarker of infection in adults as well as fetuses and neonates [11, 12]. However, detecting infection by measuring neonate CRP levels is often unsatisfactory. For example, analysis of blood CRP concentration in the umbilical cord is less sensitive (50%) when detecting antenatal infection [13] because results are influenced by fetal immaturity as well as pregnancy-related factors such as gestational age, birth weight, corticosteroid use, meconium aspiration, and noninfectious factors during the perinatal period [14–16]. In addition, fetal infection may progress rapidly to sepsis (within 24 h of birth), which might occur before serum CRP elevation is detectable [12, 17]. Therefore, new serum biomarkers are needed for the early diagnosis of fetal infection.

In our previous study, leucine-rich alpha-2 glycoprotein (LRG) was identified as an inflammatory biomarker through the semiquantitative proteomic analysis of sera in patients with rheumatoid arthritis before and after anti-tumor necrosis factor (TNF) therapy [18]. LRG has been reportedly effective in evaluating the activity of diseases such as microbial infection, rheumatoid arthritis, ulcerative colitis, and asthma [19–24]. Thus, LRG is a promising inflammatory biomarker for various inflammatory diseases; however, this factor has never been evaluated as a biomarker for fetal and neonatal infections.

The present study aimed to (1) determine the LRG levels in the umbilical cord serum of noninfected fetuses, (2) compare the efficacy of LRG with that of CRP as a biomarker for fetal infection, and (3) evaluate the underlying mechanism of elevated LRG levels in a model of fetal infection *in vitro* and *in vivo*.

## Materials and methods

### Participants and samples

The present study conformed to the Declaration of Helsinki. We obtained a written informed consent from each participant, and the study and all its protocols were approved by the Osaka University Clinical Research Review Committee (Approval Nos.: 16255, 17415, 19159).

### Participants and samples for serum LRG evaluation

We retrospectively reviewed all women who delivered at Osaka University Hospital (Osaka, Japan) between June 2012 and June 2017. We excluded multiple pregnancies, deliveries before 22 gestational weeks, and cases with lethal fetal anomaly. We then included cases of histological CAM, confirmed the diagnosis histologically by Blanc’s classification, and determined fetal infection by the histological evidence of funisitis according to the Nakayama stage system [25, 26]. These cases were classified into two subgroups: CAM with fetal infection (CAM-f[+] group, *n* = 19) and CAM without fetal infection (CAM-f[–] group, *n* = 86).

**Fig 1:**
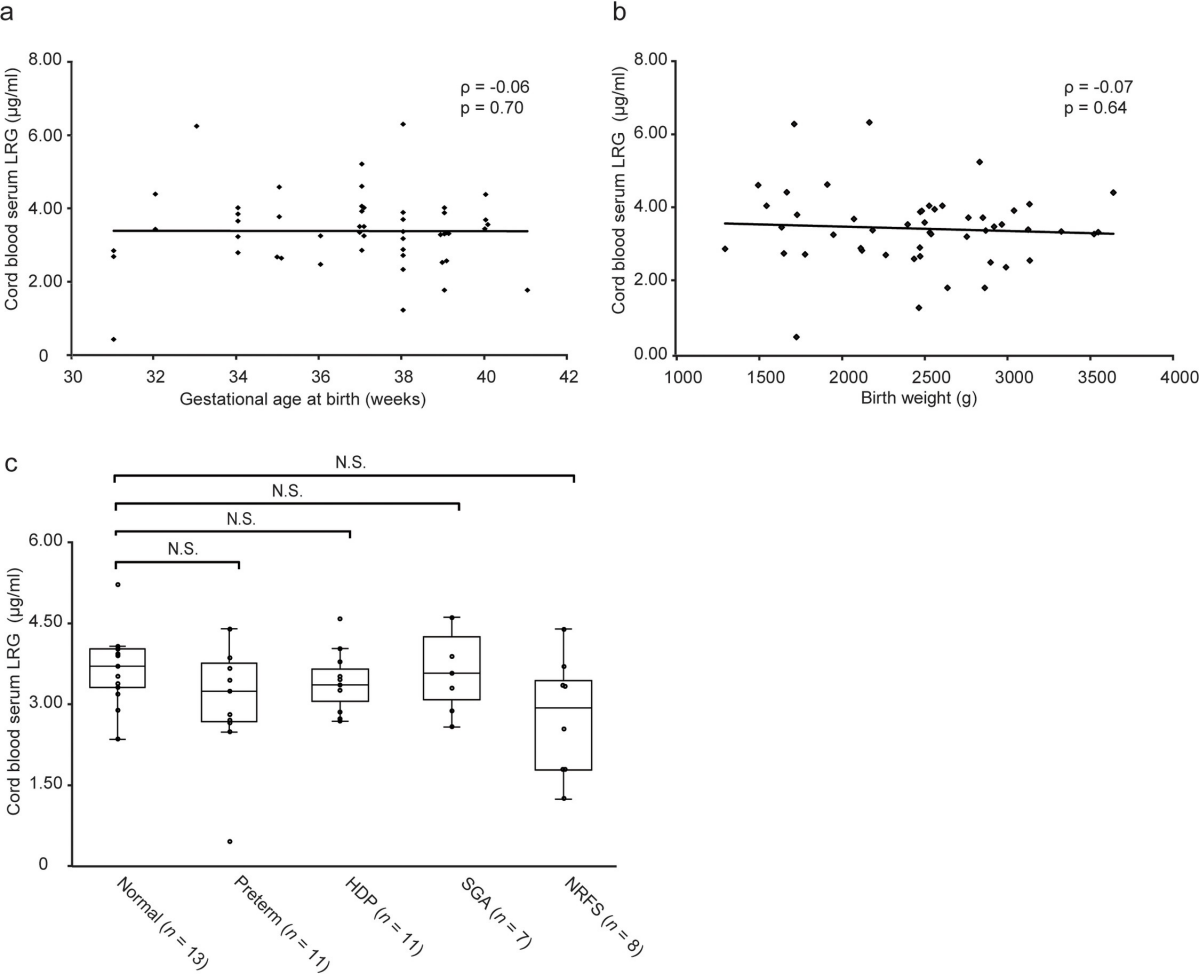
Distribution of umbilical cord serum LRG and the effect of major obstetrical/neonatal complications on LRG. **a, b,** Gestational age at birth and birth weight did not affect serum LRG levels. Spearman’s rank correlation coefficient was used to analyze the relationship. **c,** LRG levels in umbilical cord sera. The horizontal line in each box denotes the median; the box extends from 25^th^ to 75^th^ percentile of the value distribution in each group; vertically extending lines denote upper and lower adjacent values. There were no significant differences in LRG levels between normal cases and four major obstetrical/neonatal complications. Statistical significance was determined by Kruskal–Wallis test. Abbreviations: LRG, leucine-rich alpha-2 glycoprotein; Preterm, preterm birth; HDP, hypertension disorders of pregnancy; SGA, small for gestational age; NRFS, non-reassuring fetal status; N.S., not significant.

**Fig 2:**
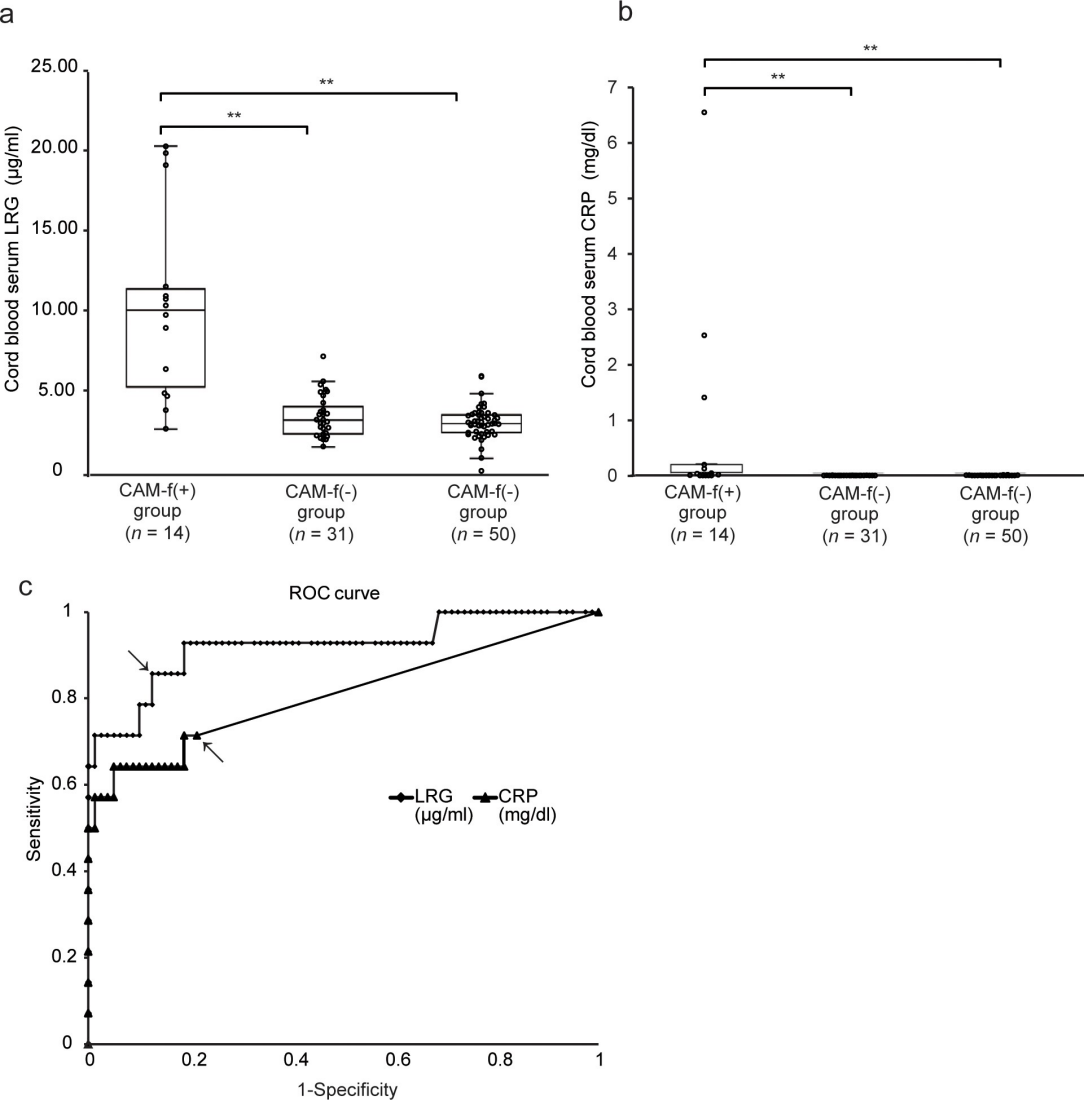
Comparison of serum LRG levels in infants with or without fetal infection. **a, b,** Levels of umbilical cord serum LRG and CRP. Within each box, the horizontal line denotes the median value; The box extends from 25^th^ to 75^th^ percentile of value distribution in each group; vertically extending lines denote upper and lower adjacent values. The CAM-f(+) group (*n =* 14) had significantly higher levels of both LRG and CRP than the CAM-f(−) (*n =* 31) and control (*n =* 50) groups. Statistical significance was determined by Kruskal–Wallis test. A double asterisk indicates *p* < 0.01. **c,** ROC curve for discriminating CAM-f(+) group (*n =* 14) and the group that combined both CAM-f(−) and control (*n =* 81) was generated to compare the usefulness of LRG (solid line) and CRP (dotted line) in predicting fetal infection. Both arrows indicate the best cutoff values. The AUC of LRG (0.92) was significantly higher than that of CRP (0.80) (*p* < 0.01). Abbreviations: LRG, leucine-rich alpha-2 glycoprotein; CRP, C-reactive protein; CAM, chorioamnionitis; N.S., not significant; ROC, receiver operating characteristic; AUC, area under the curve.

We ultimately acquired 14 and 31 umbilical cord samples from the CAM-f(+) and CAM-f(−) groups, respectively, for the LRG evaluation (Figs 1 and 2). We also selected 50 cases of patients without clinical signs of CAM who delivered at our hospital during the study period. Clinical CAM was based on the Lencki’s criteria during clinical presentation [27]. The control group included patients who had not undergone pathological evaluation. Background data were retrieved from the hospital database. A gestational age at birth of <37 weeks indicated preterm birth. Cases of hypertensive disorders of pregnancy (HDP) were diagnosed according to the criteria of the International Society for the Study of Hypertension in Pregnancy [28]. Based on Japanese birthweight percentiles, small for gestational age (SGA) was defined as a body weight below the 10^th^ percentile for gestational age and gender. Furthermore, non-reassuring fetal status (NRFS) was diagnosed according to the categories of the Eunice Kennedy Shriver National Institute of Child Health and Human Development [29].

After acquiring the informed consents, we collected samples of umbilical cord serum from each woman during birth. Umbilical cord blood was extracted by venipuncture of the umbilical vein immediately after birth and stored in blood collection tubes. The samples were then centrifuged (3,000 rpm, 4°C, 10 min), and the serum was stored in new tubes at −80°C until analysis.

### Participants and samples for histopathological evaluation

**Fig 3:**
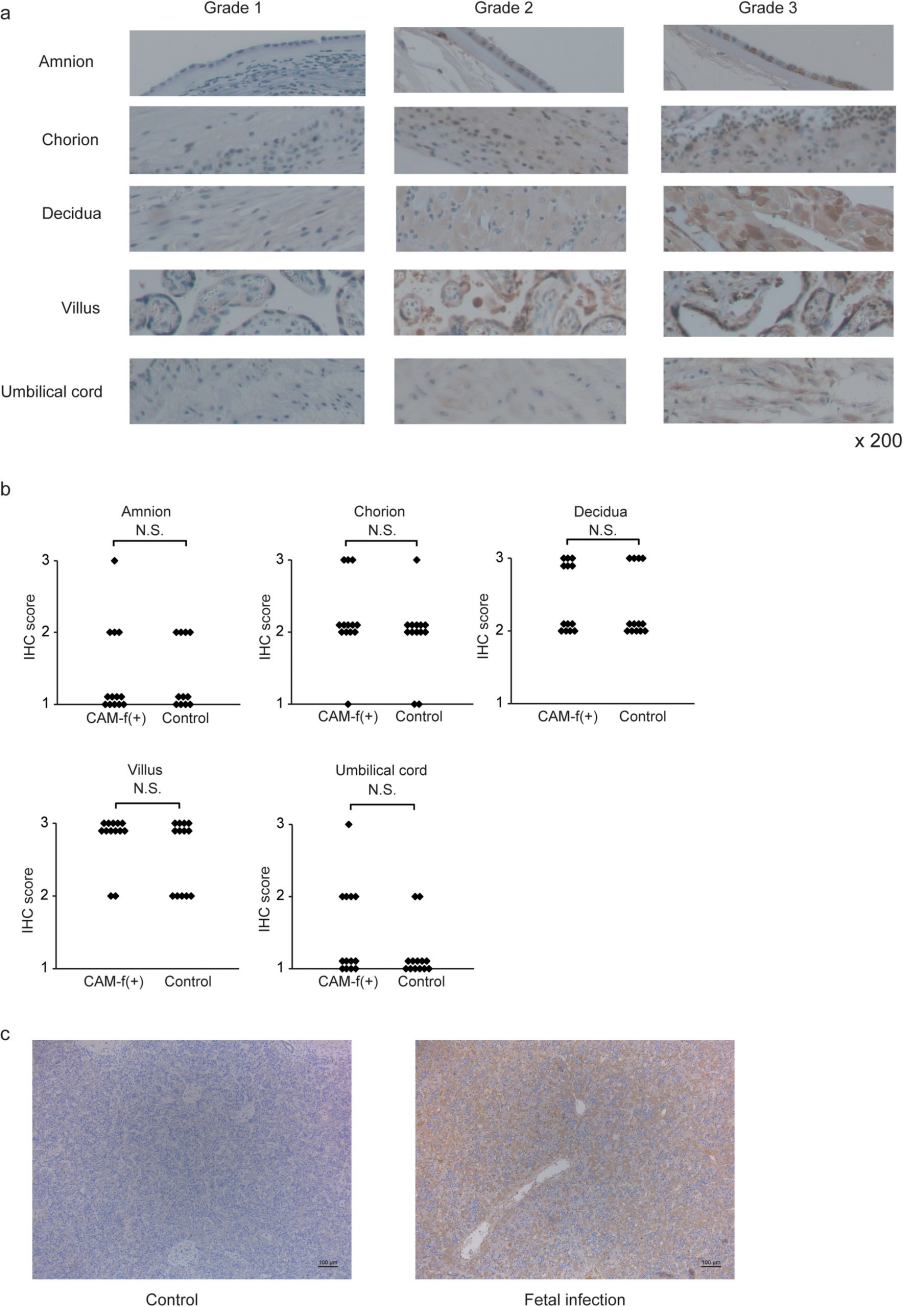
Expression of LRG in CAM-f(+) patients. **a,** Representative images of various intensities of IHC staining for LRG in tissue specimens. ×200 magnification **b**, The graphs represent the LRG expression scores in the tissue samples with or without funisitis. Statistical significance was determined by the Mann–Whitney U-test. **c,** Staining intensity of LRG in hepatocytes of autopsy tissue were stronger in the infected fetus than in the control fetus. Scale bar: 100 μm. Abbreviations: LRG, leucine-rich alpha-2 glycoprotein; CAM, chorioamnionitis; IHC, immunohistochemistry; N.S., not significant.

To histopathologically evaluate the placenta and cord (Fig 3A and 3B), we used residual formalin-fixed tissues prepared for clinical pathological examination. From the CAM-f(+) group, 13 samples were available for an additional histopathological evaluation. Regarding the control, 13 patients without pathological abnormalities were selected from all the patients who underwent pathological examination during the study period.

### Participants and samples for evaluating LRG expression in the fetal liver

The LRG expression in the fetal liver was evaluated by immunohistochemistry (Fig 3C) using autopsy samples from two cases of pregnant termination. One fetus was diagnosed with intrauterine infection caused by preterm premature rupture of membranes and severe CAM, while the other was diagnosed with bilateral renal agenesis without fetal infection. Both fetuses were terminated at 21 gestational weeks, and the disease cause was determined by autopsy. Residual formalin-fixed tissues were then used.

### Quantification of leucine-rich alpha-2 glycoprotein and C-reactive protein

Concentration of LRG was measured using a sandwich enzyme-linked immunosorbent assay (ELISA) as previously described [21, 23]. Monoclonal antibodies specific for human LRG (capture antibody; huLRB0091 and detection antibody; rbLRB0048) and mouse LRG (capture antibody; mLRA0010 and detection antibody; rLRA0094) were used to detect LRG. Furthermore, 96-well microtiter plates were coated with capture antibody then blocked with 10 mM Tris-HCl, 150 mM NaCl, pH 7.5, 0.01% Tween-20 (TBS-T) containing 0.5% bovine serum albumin, and Block Ace (DS Pharma Biomedical, Osaka, Japan). Serum (100 μl) was added to the plate and the plate incubated for 1 h. After washing, plates were incubated with detection antibody, followed by peroxidase-conjugated anti-rabbit immunoglobulin G (IgG; Southern Biotech, AL, USA). The standard curve was constructed by analyzing serial dilutions of recombinant human and mouse LRG. Human CRP concentrations were measured by the SRL clinical examination service (Osaka, Japan).

### Immunohistochemistry

Human tissue 4-μm-thick sections (placenta and cord, fetal liver) were cut from paraffin blocks, then dewaxed and rehydrated. Rabbit antihuman LRG polyclonal antibody (HPA001888, Atlas Antibodies AB, Stockholm, Sweden, 1:1000) was used as the primary antibody and samples were visualized using EnVision ChemMate (Dako, Glostrup, Denmark) according to the manufacturer’s protocol. For analyses of mouse sections, dewaxed and rehydrated sections (4 μm) were incubated for 20 min in citrate buffer (10 mM citric acid, pH 6.0) at 95–100°C for antigen retrieval. Sections were then treated with 0.3% H_2_O_2_ and blocked using Blocking One reagent (Nacalai Tesque), followed by incubation with rabbit anti-mouse LRG1 polyclonal antibody (R322, Immuno-Biological Laboratories Co. Ltd., Gunma, Japan, 1:1,000) overnight at 4°C. After washing, sections were treated with the VECTASTAIN ABC Rabbit IgG Kit (Vector Laboratories, Burlingame, CA, USA). All sections were counterstained with hematoxylin.

**Fig 4:**
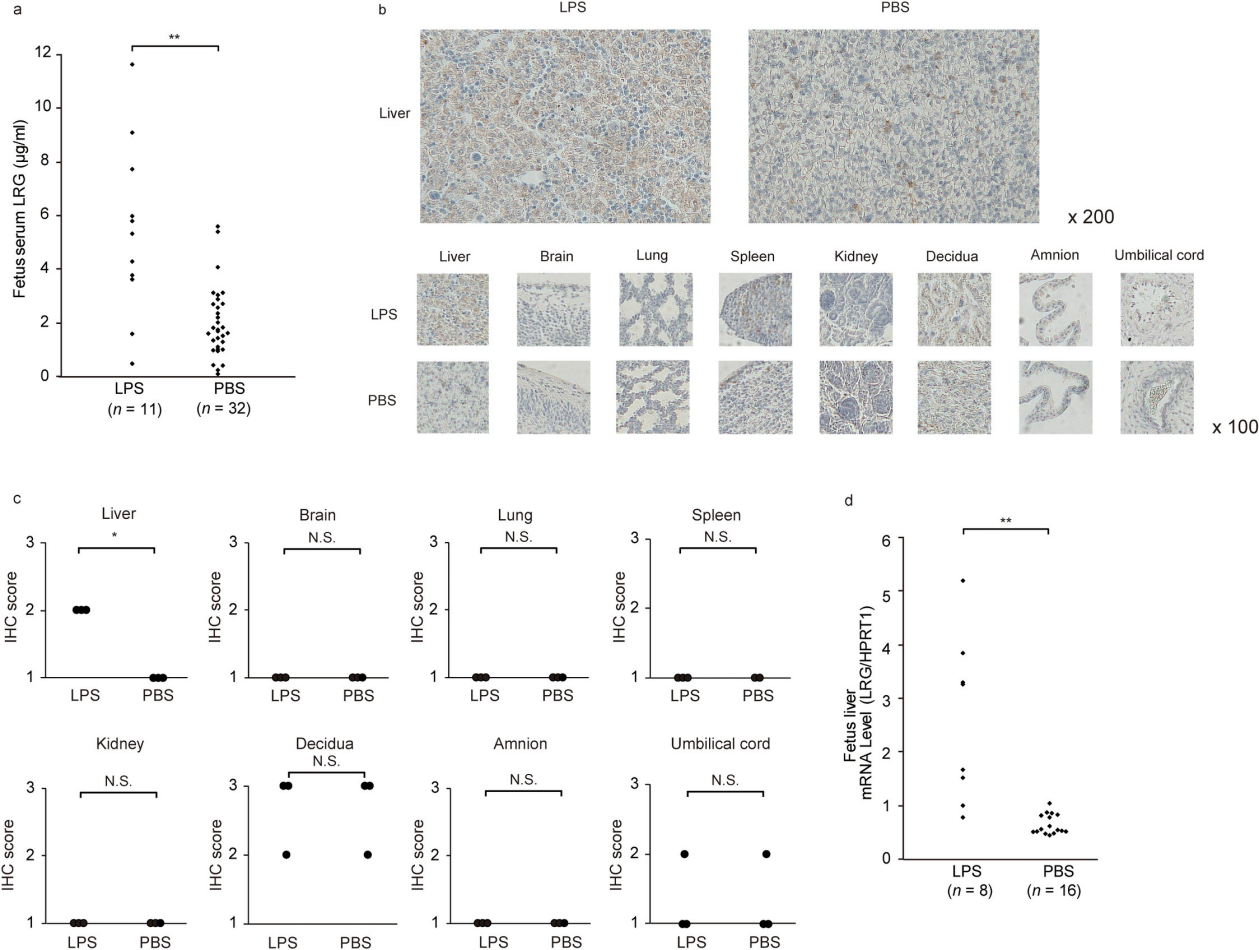
Expression of LRG in the CAM murine model. **a,** The serum LRG levels were higher in the fetuses of CAM model (*n =* 11) than in the control (*n =* 32). Statistical significance was determined by unpaired Student *t* test. **b,** Representative images of various intensities of IHC staining for LRG in tissue specimens. ×200 magnification (upper), ×100 magnification (bottom). **c,** The graphs represent the LRG expression scores in the fetuses tissue of CAM model and control. Statistical significance was determined by the Mann-Whitney U-test. A asterisk indicates *p* < 0.05. N.S., not significant. **d,** The LRG mRNA expression in fetal livers in the CAM model was significantly higher than that in the control. Statistical significance was determined by unpaired Student *t* test. A double asterisk indicates *p* < 0.01. N.S., not significant. Abbreviations: LRG, leucine-rich alpha-2 glycoprotein; CAM, chorioamnionitis; IHC, immunohistochemistry; LPS, lipopolysaccharide; PBS, phosphate-buffered saline; HPRT1, hypoxanthine phosphoribosyltransferase 1.

Independent obstetricians (Fig 3B) [E.K. & Ki.Y. & S.M.], Fig 4C [E.K. & M.E. & Ka.Y.]), who were blinded to the histological data, analyzed the stained sections using an Olympus BH2 microscope for Figs 3B and 4C. The intensity of immunostaining was graded as 1 (weak staining), 2 (moderate), or 3 (strong). Grading was determined as the highest grade identified by sampling of 5–10 random fields for each tissue.

### Murine model of chorioamnionitis at term

All experiments were performed in accordance with the National Institutes of Health guidelines for laboratory animals and with approval from the Osaka University Institutional Animal Care and Use Committee (approval #J006265). We used mouse models of localized intrauterine inflammation [30, 31] established *via* intrauterine injection of lipopolysaccharide (LPS). These stimulations caused fetal inflammation and adverse neurological and respiratory outcomes, similar to fetal infection [31, 32].

We purchased CD-1 pregnant mice from Charles River Laboratories (Kanagawa, Japan). Mice were maintained under specific pathogen-free conditions and were used for experiments at 17 or 18 days of gestation. Mouse E17 and E18 embryos from time-dated pregnant CD-1 dams were used for protein assays and mRNA analysis, respectively. Mice were anesthetized using isoflurane for minilaparotomy and infusion of either 50 μg LPS (L2880, Escherichia coli LPS, Sigma, St. Louis, MO) in 100 μL phosphate-buffered saline (PBS) or an equal volume of PBS into the uterus between the 1^st^ and 2^nd^ bilateral gestational sac closest to the cervix. Routine closure was performed, and dams allowed to recover in individual cages. Every hour up to 3 hours after surgery, we monitored respiratory rate, activity, sign of pain as writhing.

Six hours after intrauterine injection into E18 embryos, the mice were euthanized by an overdose of isoflurane and these fetal livers were excised and submerged in a collection tube containing RNAlater RNA Stabilization Reagent (QIAGEN, Valencia, CA). For E17 embryos, after 24 h of stimulation, fetal blood and tissues (brain, lungs, liver, spleen, kidneys, and placenta) were collected from surviving mice. Serum samples were separated by centrifugation and stored at −80°C until analysis. Tissues were fixed with 10% neutral buffered formalin.

### Cell lines and culture

Human hepatoma Hep3B cells were obtained from the Cell Resource Center for Biomedical Research (Tohoku University, Sendai, Japan). Human fetal hepatocyte cell line WRL68 cells were obtained from the American Type Culture Collection (Manassas, VA, USA). The Hep3B cells were cultured in Dulbecco’s Modified Eagle Medium (DMEM) while WRL68 cells (American Type Culture Collection) were cultured in Eagle’s Minimum Essential Medium. All media were supplemented with 10% fetal bovine serum (FBS; Serum Source International, Charlotte, NC, USA) with 100 U/ml penicillin and 100 μg/ml streptomycin (Nacalai Tesque, Kyoto, Japan). Cultures were maintained at 37°C in a humidified 5% CO_2_ atmosphere. The identity of each cell line was confirmed by DNA fingerprinting using short tandem repeat profiling, as previously performed [33]. Mycoplasma testing was routinely performed by MycoAlert Mycoplasma Detection Kit (Lonza, Rockland, ME).

### Luciferase assay

Human LRG promoter sequences (−585/+78 relative to the predicted transcription start site according to the expressed sequence tag database) were amplified by polymerase chain reaction (PCR) using human genomic DNA (69237; Novagen) and were cloned into a pGL3 luciferase vector (Promega). We transiently transfected Hep3B cells with the pGL3 vector containing the human LRG promoter and the control pRL-TK vector (Promega) using Lipofectamine 2000 reagent (Invitrogen) according to the manufacturer’s instructions. Twelve hours after transfection, cells were starved by incubating in DMEM containing 1% fetal calf serum for 2 h, then stimulated with 10 ng/ml of various cytokines (interleukin [IL]-1β, IL-6, IL-22, interferon [IFN]-γ, and TNF-alpha [α]; obtained from Peprotech, Rocky Hill, NJ) for 6 h. Luciferase activity was determined using a dual-luciferase assay system (Promega) according to the manufacturer’s instructions. Relative firefly luciferase activity was determined after normalization with Renilla luciferase activity. This assay was performed in triplicate.

### Western blotting

We stimulated WRL68 cells with 100 ng/ml cytokines (IL-6, IL-1β, TNF-α) and 0.5% FBS. For western blotting, cells were stimulated for 15 min and lysed using radioimmunoprecipitation assay buffer (10 mM Tris-HCl, pH 7.5, 150 mM NaCl, 1% Nonidet P-40, 0.1% sodium deoxycholate, 0.1% sodium dodecyl sulfate, 1 protease inhibitor cocktail [Nacalai Tesque]) and 1 phosphatase inhibitor cocktail (Nacalai Tesque), followed by centrifugation (13,200 rpm, 4°C, 15 min) to collect the supernatants. Extracted proteins were subjected to sodium dodecyl sulfate-polyacrylamide gel electrophoresis as previously described [34]. Samples were transferred onto polyvinylidene difluoride membranes and treated with several antibodies: anti-phospho-signal transducer and activator of transcription 3 (STAT3) antibodies (#9145, Cell Signaling Technology, Danvers, MA, USA), anti-total STAT3 antibodies (sc-482, Santa Cruz Biotechnology, Dallas, TX, USA), anti-phospho-nuclear factor kappa-light-chain-enhancer of activated B (NF-κB) antibodies (#3033s, Cell Signaling Technology), anti-NF-κB antibodies (#8242s, Cell Signaling Technology), or anti-glyceraldehyde 3-phosphate dehydrogenase antibodies (sc-25778, Santa Cruz Biotechnology), as previously described [34].

### Quantitative real-time reverse transcription polymerase chain reaction analysis

We stimulated WRL68 cells and Hep3B with 100 ng/ml cytokines (IL-6, Il-22, IL-1β, TNF-α) for 4 h in the presence of 0.5% FBS. Cells and mouse livers were disrupted in Buffer RLT (QIAGEN, Hilden, Germany) and total RNA collected using the RNeasy Mini kit (QIAGEN). Complimentary DNA was generated using the QuantiTect Reverse Transcription Kit (QIAGEN). Real-time PCR (qPCR) was performed on the StepOnePlus Real-time PCR system (Applied Biosystems, Darmstadt, Germany) using SYBR Premix Ex Taq (Takara Bio, Shiga, Japan). Levels of target gene expression were normalized to glycerol-3-phosphate dehydrogenase (G3PDH) or murine hypoxanthine phosphoribosyltransferase 1 (HPRT1) expression in each sample [35]. Each reaction was performed in triplicate.

The following primers were designed and used for qPCR: human LRG, sense 5′-TTTACAGGTGAAACTCGGGG-3′, antisense 5′-ACCCCAAGCTAAGTGGGACT-3′; human CRP, sense 5′-GAACTTTCAGCCGAATACATCTTTT-3′, antisense 5′-CCTTCCTCGACATGTCTGTCT-3′, G3PDH, sense 5′-AGCAATGCCTCCTGCACCACCAAC-3′, antisense 5′-CCGGAGGGGGCCATCCACAGTCT-3′; murine LRG, sense 5′-ATCAAGGAAGCCTCCAGGAT-3′, antisense 5′-CAGCTGCGTCAGGTTGG-3′; murine HPRT1, sense 5′-TCAGTCAACGGGGGACATAAA-3′, antisense 5′-GGGGCTGTACTGCTTAACCAG-3′.

### Statistical analysis

Statistical analyses were performed using Excel Statistics (Social Survey Research Information, Tokyo, Japan) and JMP® 15.1.0 (SAS Institute Inc., Cary, NC, USA). Data are shown as the mean ± standard deviation for results of *in vitro* experiments and mean ± standard error of the mean for results of *in vivo* experiments. The Student t-test, Mann-Whitney U, Pearson’s chi-square, Kruskal-Wallis test followed by Steel test, One-way analysis of variance followed by Dunnett’s test were used to compare date between groups, as appropriate. Spearman’s rank correlation coefficient was used to determine the relationship between two variables. Multivariable logistic regression analysis was performed to determine the optimal cutoff level of LRG, and the receiver operating characteristic (ROC) curves and area under the ROC curve (AUC) were estimated. We considered *p* < 0.05 to be statistically significant.

## Results

### Study population

According to the pathological examination of the placenta and umbilical cord, 45 cases were confirmed as CAM with or without fetal infection (CAM-f[+] group [*n =* 14], CAM-f[−] group [*n =* 31]). Cases of preterm birth, SGA, or NRFS did not overlap. The control group (*n =* 50) included participants with four major obstetrical/neonatal complications, namely, preterm birth (“preterm”) (*n =* 11), HDP (*n =* 11), SGA (*n =* 7), and NRFS (*n =* 8); the 13 remaining cases had no obstetric or maternal complications (“normal”). Table 1 summarizes the patient characteristics, and Table 2 presents the relationship of patient characteristics with CAM-f(+). According to the multivariable analysis, CAM with fetal infection was associated with elevated LRG concentration but not with other variables, such as CRP, maternal age, gestational age at birth, mode of delivery, and Apgar score.

**Table 1:** Patients characteristics of the control group.

		Normal	Preterm	HDP	SGA	NRFS	*p* value
		(*n =* 13)	(*n =* 11)	(*n =* 11)	(*n =* 7)	(*n =* 8)	
**Maternal age**							
median, years		34.0	33.0	37.0	35.0	37.5	0.53
IQR, years		32.0–35.0	31.0–38.0	31.0–39.0	31.0–35.5	34.5–39.3	
**GA at birth**							
median, weeks		38.0	34.0	36.0	39.0	39.0	< 0.01
IQR, weeks		37.0–38.0	32.0–34.0	35.0–37.0	37.5–39.0	39.0–40.0	
Post hock test, *p* value		Reference	< 0.01	0.04	0.50	<0.01	
**Mode of delivery**							
CD, n (%)		9 (69.2)	6 (54.5)	4 (36.4)	3 (42.9)	8 (100)	0.053
**Birth weight**							
median, g		2768	1954	2190	2440	2871	< 0.01
IQR, g		2564–3044	1723–2302	1602–2734	2144–2496	2803–3188	
Post hock test, *p* value		Reference	< 0.01	0.07	0.01	0.70	
**Apgar Score**							
1min	median	8.0	8.0	8.0	8.0	7.0	0.01
	IQR	8.0–8.0	8.0–8.0	8.0–8.0	7.5–8.0	5.0–7.3	
Post hock test, *p* value		Reference	0.46	0.86	0.38	< 0.01	
5min	median	9.0	9.0	9.0	9.0	9.0	0.23
	IQR	9.0–9.0	8.5–9.0	9.0–9.0	9.0–9.0	7.3–9.0	

Median (IQR) or number (percentage per column) is shown. Data were analyzed by the Kruskal-Wallis test followed by Steel test, and the Pearson chi-square test followed by Bonferroni correction.

Abbreviations; Preterm, preterm birth; HDP, hypertension disorder of pregnancy; SGA, small for gestational age; NRFS, non-reassuring fetal status; IQR, Interquartile range; GA, gestational age; CD, cesarean delivery.

**Table 2:** Relation of patients characteristics to CAM-f(+).

		CAM-f(+) (*n =* 14)	CAM-f(−) (*n =* 31)	Control (*n =* 50)	*p* value	Multivariable analysis *p*-value
**LRG**						
median, μg/ml		10.37	3.61	3.39	< 0.01	< 0.01
IQR, μg/ml		5.21–13.7	2.71–4.65	2.81–3.93		
post hock test, *p*-value		< 0.01	0.54	Reference		
**CRP**						
median, mg/dl		0.28	0.01	0.01	< 0.01	0.70
IQR, mg/dl		0.01–0.50	0.01–0.01	0.01–0.01		
post hock test, *p*-value		< 0.01	0.87	Reference		
**Maternal age**						
median, years		32.5	32.0	35.0	0.39	0.38
IQR, years		30.3–37.3	29.0–37.0	31.3–38.0		
**GA at birth**						
median, weeks		39.5	36.0	37.0	< 0.01	0.73
IQR, weeks		38.3–40.0	34.5–38.0	35.0–39.0		
post hock test, *p*-value		< 0.01	0.43	Reference		
**Mode of delivery**						
CD, n (%)		2 (14.3)	8 (25.8)	30 (60.0)	< 0.01	0.10
post hock test, *p*-value		< 0.01	0.43	Reference		
**Birth weight**						
median, g		3160	2310	2496	< 0.01	0.11
IQR, g		2806–3279	1945–2466	2088–2873		
post hock test, *p*-value		< 0.01	0.12	Reference		
**Apgar Score**						
1min	median	8.0	8.0	8.0	0.92	0.25
	IQR	7.3–8.0	8.0–8.0	7.3–8.0		
5min	median	9.0	9.0	9.0	0.05	0.99
	IQR	8.0–9.0	9.0–9.0	9.0–9.0		

Median (IQR) or number (percentage per column) is shown. Data were analyzed by the Kruskal-Wallis test followed by Steel test, and the Pearson chi-square test followed by Bonferroni correction. Multivariable logistic regression model was used for comparison between CAM-f(+) group and “no fetal infection” group (combining CAM-f[−] with control).

Abbreviations: CAM, chorioamnionitis; LRG, leucine-rich alpha-2 glycoprotein; IQR, Interquartile range; CRP, C-reactive protein; GA, gestational age; CD, cesarean delivery.

ELISA, which was used for quantifying the LRG levels in the control group, revealed that umbilical cord serum LRG was not affected by gestational age at delivery (*ρ* = -0.06, *p* = 0.70) nor neonatal birth weight (*ρ* = -0.07, *p* = 0.64) (Fig 1A and 1B). In addition, no significant differences were found in serum LRG concentration in terms of major obstetric complications (*p* = 0.27; Fig 1C). In the control group, the mean serum LRG concentration obtained from the umbilical cord was 3.39 (2.81–3.93) μg/ml, which was substantially lower than that in healthy adults analyzed in our previous study [20].

As shown in Fig 2A, the median (IQR) serum LRG concentration in the CAM-f(+) group was 10.37 (5.21–13.7) μg/ml, which was significantly higher than that of the CAM-f(−) or control groups (3.61 [2.71–4.65] μg/ml, *p* < 0.01; 3.39 [2.81–3.93] μg/ml, *p* < 0.01). The median LRG values were not significantly different between the CAM-f(−) and control groups (*p* = 0.57). The highly sensitive CRP was also significantly elevated in the CAM-f(+) group (0.28 [0.01–0.50] mg/dl; *p* < 0.01; Fig 2B) compared with that in other groups.

Considering that the LRG and CRP values were not significantly different between the CAM-f(−) group and the control group, we combined these two groups into one group (termed as “no fetal infection”) to compare with the CAM-f(+) group (“fetal infection”). In the ROC analyses, LRG (0.92, 95% confidence interval [CI], 0.82–1.02) had a higher AUC than CRP (0.80, 95% CI, 0.66–0.96; Fig 2C). The cutoff value was 5.08 μg/ml for serum LRG and 0.006 mg/dl for serum CRP. In addition, the sensitivity and specificity of LRG were 86% and 88%, respectively, which were higher than those of CRP (71% and 81%, respectively). Both the positive predictive value (PPV, 0.55) and negative predictive value (NPV, 0.97) of LRG were also higher than those of CRP (0.4 and 0.94, respectively).

### Fetal liver is the main source of LRG in umbilical cord serum during fetal infection

Local LRG expression is the source of increased serum LRG in patients with ulcerative colitis [22]. Therefore, we investigated whether LRG is elevated locally in the inflamed placenta or umbilical cord. Fig 3A presents the representative images of LRG immunostaining for the amnion, chorion, decidua, villus, and umbilical cord. Table 3 summarizes the patient characteristics. The overall immunostaining scores for these tissues were not significantly different between the CAM-f(+) and control (Fig 3B, all, *p* > 0.05).

**Table 3:** Patients characteristics for histopathological evaluation.

		CAM-f(+)	Control	
		(*n =* 13)	(*n =* 13)	*p* value
**Maternal age**				
median, years		34.0	33.0	0.90
IQR, years		30.0–37.0	30.0–38.0	
**GA at birth**				
median, weeks		40.0	38.0	0.11
IQR, weeks		38.0–41.0	37.0–39.0	
**Mode of delivery**				
CD, n (%)		5 (38.5)	5 (38.5)	1.00
**Birth weight**				
median, g		3104	2652	0.07
IQR, g		2786–3196	2532–3016	
**Apgar Score**				
1min	median	8.0	8.0	0.86
	IQR	7.0–8.0	8.0–8.0	
5min	median	9.0	9.0	0.82
	IQR	8.0–9.0	9.0–9.0	

Median (IQR) or number (percentage per column) is shown. Data were analyzed by Mann-Whitney U test and the Pearson chi-square test. Abbreviations: CAM, chorioamnionitis; IQR, Interquartile range; GA, gestational age; CD, cesarean delivery.

LRG expression is reportedly upregulated in the liver during the acute-phase response [36]. Thus, hepatocytes might produce LRG, ultimately causing elevation in serum LRG levels in the fetus. In the autopsy specimen analysis, the fetal infection case had a stronger LRG expression in the hepatocytes than the case without fetal infection (Fig 3C).

### Fetal liver is the primary source of fetal serum LRG in a mouse model of CAM

The number of parturient mice was significantly higher in the CAM model than in the PBS-administered control (CAM model, 52%; control, 15%; *p* < 0.01). Meanwhile, the frequency of intrauterine fetal demise at 24 h after intrauterine injection was also significantly higher in the CAM model than in the control (CAM model, 34%; control, 6.5%; *p* < 0.01).

At 24 h after injection, fetal serum LRG levels were significantly elevated among fetuses of the CAM model (*n =* 11) compared with those among control fetuses (*n =* 32) (Fig 4A; 5.36 ± 3.09 versus 1.96 ± 1.30, *p* < 0.01). As shown in Fig 4B, the LRG expression was stronger in the hepatocytes of CAM-model fetuses than in those of the control fetuses; nonetheless, the positive staining of granulocytes scattering in the liver of both groups was equal. In the fetal liver section, the immunostaining scores were moderate and were significantly higher in CAM-model fetuses (*n* = 3) than in the control (*n* = 3) (Fig 4C, *p* = 0.03). In contrast, brain, lung, spleen, kidney, amnion, decidua, or umbilical cord tissues had a no significantly difference of LRG expression between CAM-model fetuses and control. Meanwhile, the liver tissues of CAM-model fetuses (*n =* 8) had significantly higher levels of mRNA than the control fetuses (*n =* 16) (Fig 4D, *p* < 0.01).

### Fetal hepatocytes produce LRG in vitro under inflammatory conditions

**Fig 5:**
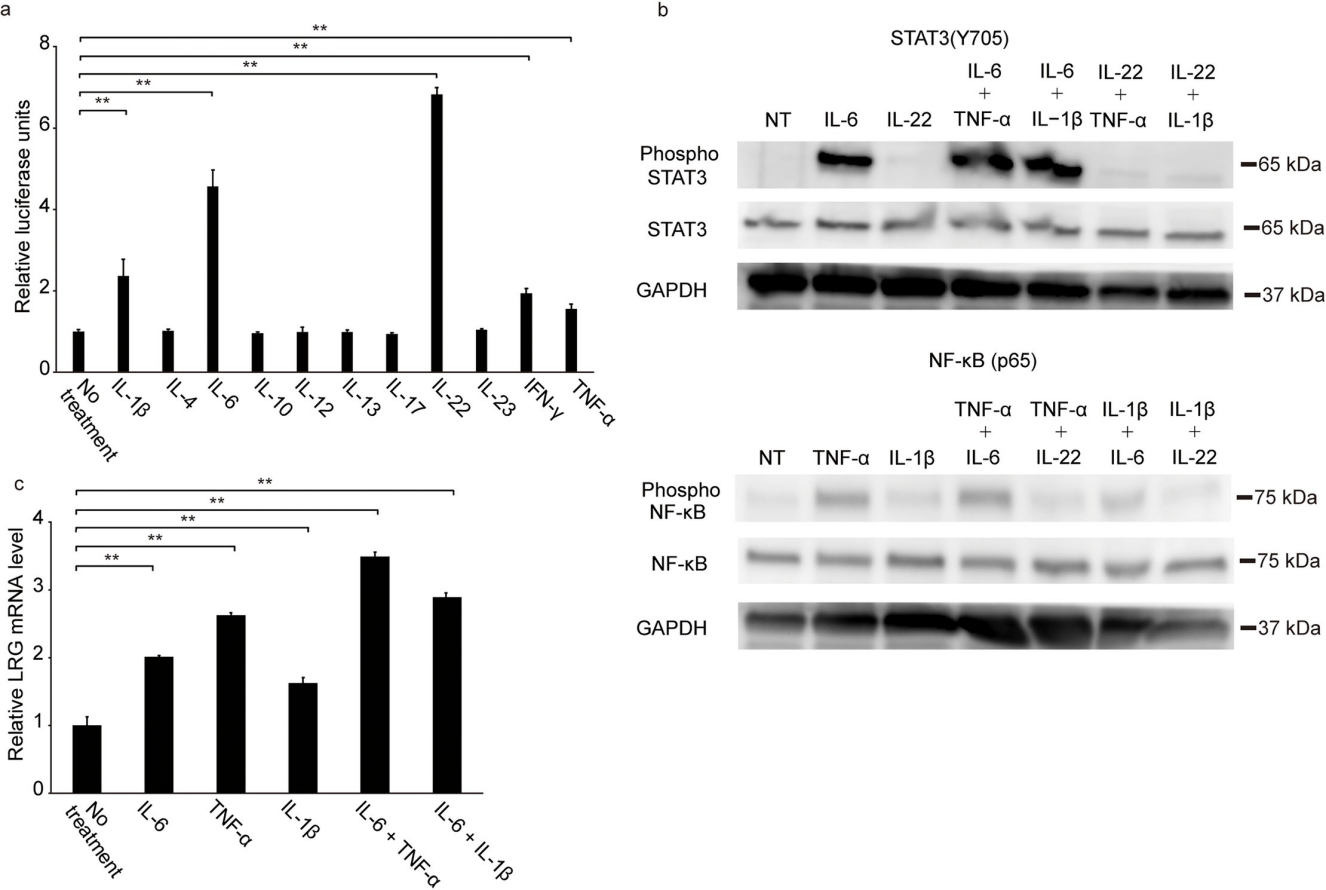
Expression of LRG in human hepatocyte-stimulated proinflammatory cytokines. **a,** Luciferase reporter assay with Hep3B showed that LRG could be induced by IL-1β, IL-6, IL-22, IFN-γ, and TNF-α. **b,** STAT3 (Y705) phosphorylation was induced in the cytoplasm of WRL68 cells stimulated by IL-6. The phosphorylation in IL-22 stimulation was extremely weak. TNF-α induced the phosphorylation of NF-κB (p65) in WRL68 cells more strongly than IL-1β. GAPDH was used as a control of relative amounts of proteins in each sample. **c,** LRG mRNA expressions in WRL68 stimulated with proinflammatory cytokines. The expression levels of LRG mRNA were corrected with those of G3PDH mRNA. Values are the means ± SD of three independent determinations. The data were analyzed by an ANOVA followed by Dunnett’s analysis. A double asterisk indicates *p* < 0.01. Abbreviations: LRG, leucine-rich alpha-2 glycoprotein; IL-1β, interleukin-1β; IL-4, interleukin-4; IL-6, interleukin-6; IL-10, interleukin-10; IL-12, interleukin-12; IL-13, interleukin-13; IL-17, interleukin-17; IL-22, interleukin-22; IL-23, interleukin-23; IFN-γ, interferon-γ; TNF-α, tissue necrosis factor-alpha; STAT3, signal transducer and activator of transcription 3; NF-κB, nuclear factor-kappa B; GAPDH, glyceraldehyde-3-phosphate dehydrogenase; ANOVA, analysis of variance.

The luciferase reporter assay revealed that relative luciferase units were increased in Hep3B cells after IL-1β, IL-6, IL-22, IFN-γ, and TNF-α stimulation (Fig 5A, *p* < 0.01). Among these cytokines, IL-1β, IL-6, and TNF-α are associated with intrauterine infection [37–39]. Western blot analysis revealed that in WRL68 cells, IL-6 stimulation induced STAT3 phosphorylation, while TNF-α and IL-1β stimulation induced NF-κB phosphorylation (Fig 5B). However, IL-22, which was the most potent inducer of LRG promoter activation in Hep3B cells, failed to activate STAT3 in WRL68 cells (Fig 5B). Therefore, we focused on the role of IL-1β, IL-6, and TNF-α in inducing LRG in WRL68 cells. The qPCR analysis revealed that these cytokines significantly induced the expression of *LRG* mRNA (*p* < 0.01) (Fig 5C). Induction of LRG expression was higher after stimulation with both IL-6 and TNF-α than with IL-6 or TNF-α alone (*p* < 0.01). Notably, consistent with the previous report [40], *CRP* mRNA expression was undetectable in WRL68 cells even after cytokine stimulation, although it was upregulated by IL-6 in Hep3B cells (S1 Fig).

## Discussion

The present study revealed LRG elevation in fetal infection. Notably, assessment of umbilical cord serum LRG could possibly detect fetal infection in CAM cases. Thus, our findings provide information helpful for improving the detection rate of fetal infection.

In adults, CRP, procalcitonin (PCT), and IL-6 are considered useful APRs. CRP measurement is simple, often automated, and inexpensive; thus, CRP is the most commonly measured APR for detecting infection [41, 42]. PCT and IL-6 are also often examined in cases wherein a more severe infection, such as sepsis, is suspected because these markers are reportedly better in detecting sepsis than CRP [41–43]. These biomarkers are also reportedly useful for detecting fetal infection [44]. However, since the late 1990s, these biomarkers clearly have not provided adequate information regarding their ability in detecting infections in fetuses [12], and they have been poorly utilized in the context of fetal infection in which several explanations have been proposed.

The acute-phase protein CRP is released from the liver during inflammation. However, CRP is minimally produced in fetuses [40]. This study measured the mRNA levels of the *CRP* gene in Hep3B cells derived from a human adult liver and WRL-68 cells derived from a fetal liver. We found that fetal cells do not produce CRP after stimulation with any cytokines. The authors proved that hepatocyte nuclear factor 1 (HNF-1), which is a transcription factor critical for *CRP* gene expression, is not expressed in fetal hepatocytes. Thus, fetal liver immaturity may lead to low HNF-1 expression in hepatocytes, resulting in an insufficient elevation of CRP for detecting fetal infection [11, 13, 45], as supported by our data of *CRP* mRNA levels (S1 Fig).

In the present study, the increased serum LRG levels detected in fetal infection were attributable to fetal liver cells rather than the placenta and umbilical cord (Fig 3B and 3C). The expression of LRG in fetal liver cells was not evaluated previously. Contrary to CRP [40], LRG upregulation has been observed in fetal liver-derived WRL-68 cells in response to cytokine stimulation (Fig 5), consistent with our study data. Thus, LRG is produced in human and mouse fetal hepatocytes under inflammatory conditions *in vivo* (Figs 3 and 4). The difference between the induction of CRP and LRG expression in fetal hepatocytes highlights the utility of LRG as a marker for detecting fetal infection.

Both PCT and IL-6 have been suggested to be biomarkers for severe fetal infection such as neonatal sepsis [13]. However, levels of these ARPs in neonates, as well as CRP, are affected by physiological and/or pathological conditions during pregnancy [14, 44, 46]. For example, a study on serum PCT values in 121 healthy, full-term infants without infection revealed that the mean PCT value at birth was 0.094 μg/L, increasing to 2.47 μg/L (*p* < 0.01) and 0.83 μg/L (*p* < 0.01) at 24 and 48 h after birth, respectively [47]. Therefore, the PCT expression in fetuses and neonates is not regulated by infectious stimuli but most likely by physiological factors during the perinatal period. IL-6 is reportedly related to maternal preeclampsia and HDP accompanied by proteinuria or maternal organ damage [48]. This previous study examined 43 neonates born to 19 healthy mothers and 24 mothers with preeclampsia and found that the umbilical cord IL-6 level was significantly higher among infants of mothers with preeclampsia (114.51 pg/ml) compared with that among infants of healthy mothers (23.72 pg/ml; *p* < 0.01). Similarly, CRP values are readily influenced by noninfectious factors during the perinatal period [14–16]. Taken together, pregnancy conditions can affect the utility of diagnostic markers in fetuses. Nevertheless, in our study, LRG was not affected by gestational age, neonatal body weight, nor major obstetrical complications (Fig 1), thereby more advantageous than other markers for fetal infection diagnosis.

To our knowledge, no previous studies have investigated the expression and regulation of LRG in human fetal hepatocytes. During pregnancy, the main cytokines that are reportedly involved in intrauterine inflammation are IL-6, which stimulates the STAT3 pathway, as well as IL-1β and TNF-α, which are major NF-κB pathway activators [37–39]. In the current study, these cytokines significantly enhanced the LRG expression in fetal liver cells (Fig 5). Moreover, a database search for transcription-factor–binding sites indicated that the LRG promoter contains two possible STAT-binding motifs and also a putative binding motif for NF-κB [49]. Thus, both the STAT3 and NF-κB pathways cause the increased production of LRG during intrauterine inflammation. This finding may be another key advantage of measuring LRG for the early diagnosis of fetal infection.

Our study is the first to demonstrate the relationship between fetal infection and serum LRG concentration from the umbilical cord. As a noninvasive biomarker, umbilical cord serum LRG is a useful parameter that enables the detection of infection during fetal stage and prevents aggravation of infection in neonates. However, our study has some limitations that should be acknowledged. First, this study is a single-center retrospective research with a small sample size; consequently, unmeasured bias may have existed. Our study possibly has sample selection bias. The effect of prematurity and noninfectious factors, such as meconium aspiration and corticosteroid use, is not sufficiently assessed in this study. Second, patients with culture-proven neonatal sepsis were not included in our analysis because early-onset neonatal sepsis is rare in our facility and did not occur during the study period. Therefore, we had not investigated the diagnostic value of LRG in the context of neonatal sepsis. According to systematic reviews, PCT [50] and IL-6 [51, 52] are potential biomarkers for neonatal sepsis, but their reported diagnostic utilities in neonatal sepsis vary between studies and remain to be vaguely established. Multicenter prospective studies are required to clarify the utility of LRG in comparison with CRP, PCT, and IL-6 in detecting fetal infection as well as neonatal sepsis.

Third, we did not explore the diagnostic utility of LRG quantification in other obstetric specimens, such as maternal serum and amniotic fluid. Several markers in maternal serum are reportedly useful for the prenatal diagnosis of intrauterine infection [53, 54]. However, our preliminary analysis suggests that LRG in maternal serum reflects maternal inflammation rather than fetal infection (S2 Fig). A larger sample size is required to analyze its utility. In addition, IL-8 [55, 56] and urocortin-1 [57–59] in amniotic fluid reportedly predict CAM and preterm delivery, respectively. However, the applicability of our assay system for LRG measurement in amniotic fluid remains unproven; thus, further research is necessary. Fourth, the underlying mechanism of LRG elevation in clinical samples remained unconfirmed. Nevertheless, we obtained *in vitro* and *in vivo* data from an animal model and autopsy cases. Based on these data, IL-6, IL-1β, and TNF-α elevation during fetal infection stimulates LRG expression in fetal hepatocytes to increase the umbilical cord serum LRG levels. We intend to prove this hypothesis in future studies.

In conclusion, LRG is an effective marker for detecting fetal infection, and fetal hepatocytes may represent the primary source of LRG *in utero*.

## Supporting information

S1 Fig LRG concentrations in maternal sera and umbilical cord sera. There was no relationship between maternal serum and umbilical cord serum LRG concentrations. Spearman’s rank correlation coefficient was used to analyze the relationship. Abbreviations: LRG, leucine-rich α2-glycoprotein.
(TIF)

S2 Fig CRP expression in Hep3B and WRL68 cell lines. **a,b** CRP mRNA expression in Hep3B (a) and WRL68 (b) cells stimulated with proinflammatory cytokines. Values are the means ± SD of three independent determinations. The data were analyzed by an ANOVA followed by Dunnett’s analysis. A double asterisk indicates *p* < 0.01. Abbreviations: LRG, leucine-rich α2-glycoprotein; IL-1β, interleukin-1β; IL-6, interleukin-6; TNF-α, tissue necrosis factor-α; GAPDH, glyceraldehyde-3-phosphate dehydrogenase; ANOVA, analysis of variance; **, *p* < 0.01.
(TIF)

S1 File Original blot images for Fig 5B. Original blot images underlying the results of Fig 5B were provided as a zip file.
(ZIP)
