# Supplementary figures and images for: Evaluation of leucine-rich alpha-2 glycoprotein as a biomarker of fetal infection
Source: PLoS One. 2020 Nov 19;15(11):e0242076. doi: 10.1371/journal.pone.0242076 (PMC7676652; doi:10.1371/journal.pone.0242076)

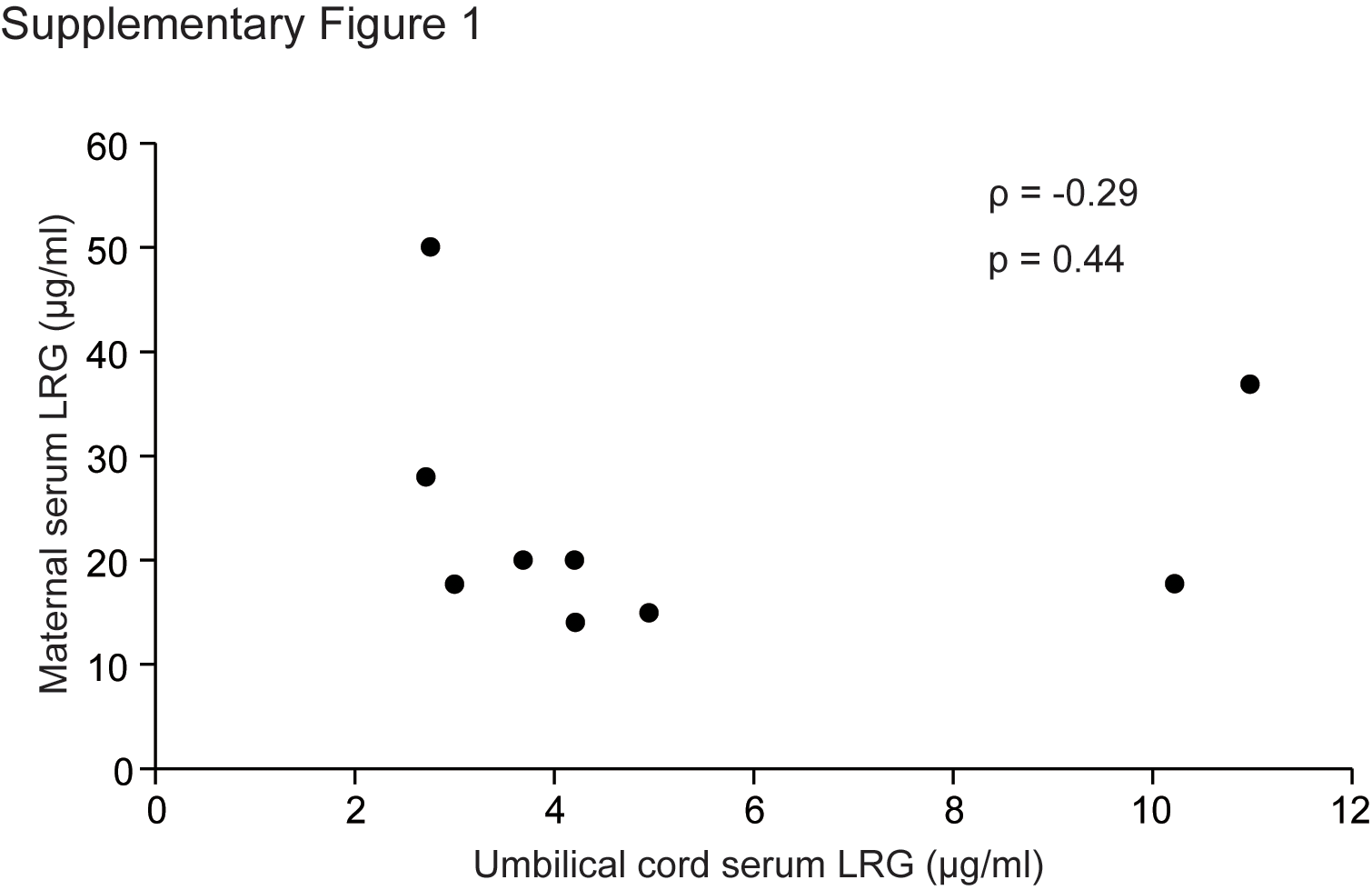

Supplement: S1 Fig — There was no relationship between maternal serum and umbilical cord serum LRG concentrations. Spearman’s rank correlation coefficient was used to analyze the relationship. Abbreviations: LRG, leucine-rich α2-glycoprotein. (TIF) [file pone.0242076.s001.tif]

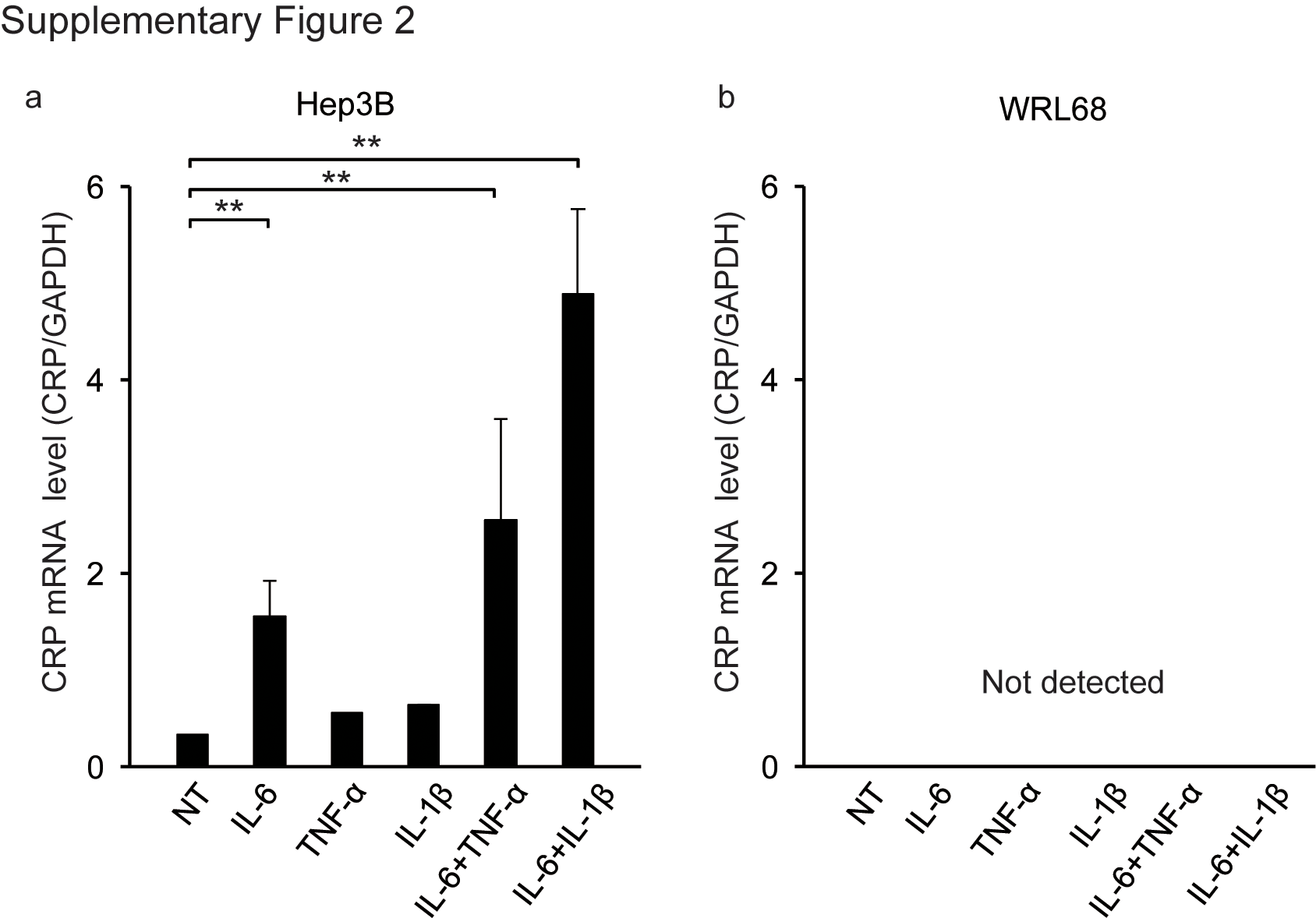

Supplement: S2 Fig — a,b CRP mRNA expression in Hep3B (a) and WRL68 (b) cells stimulated with proinflammatory cytokines. Values are the means ± SD of three independent determinations. The data were analyzed by an ANOVA followed by Dunnett’s analysis. A double asterisk indicates p < 0.01. Abbreviations: LRG, leucine-rich α2-glycoprotein; IL-1β, interleukin-1β; IL-6, interleukin-6; TNF-α, tissue necrosis factor-α; GAPDH, glyceraldehyde-3-phosphate dehydrogenase; ANOVA, analysis of variance; **, p < 0.01. (TIF) [file pone.0242076.s002.tif]

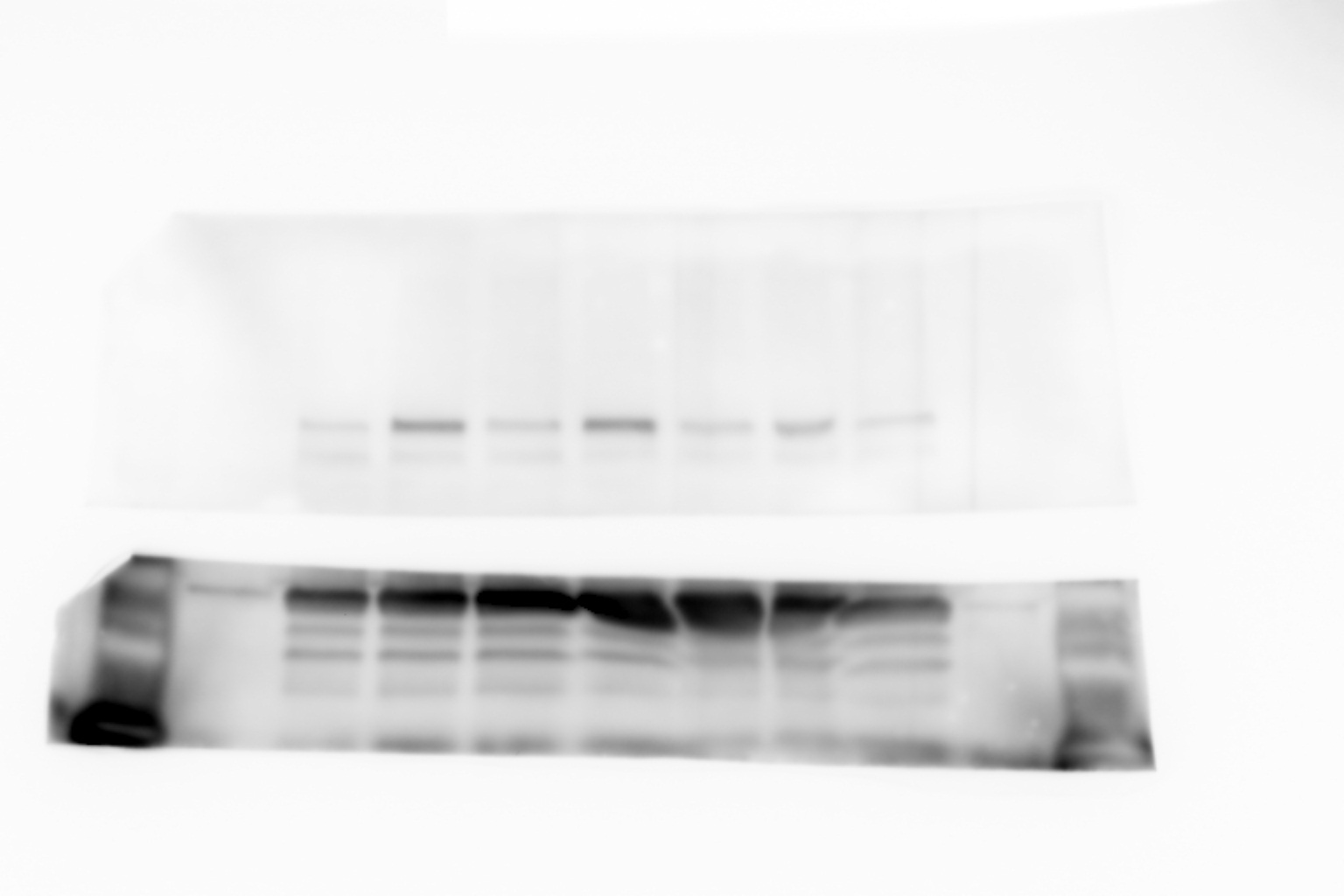

Supplement: S1 File — Original blot images underlying the results of Fig 5B were provided as a zip file. (ZIP) [file pone.0242076.s003.zip › pNFkB GAPDH.jpg]

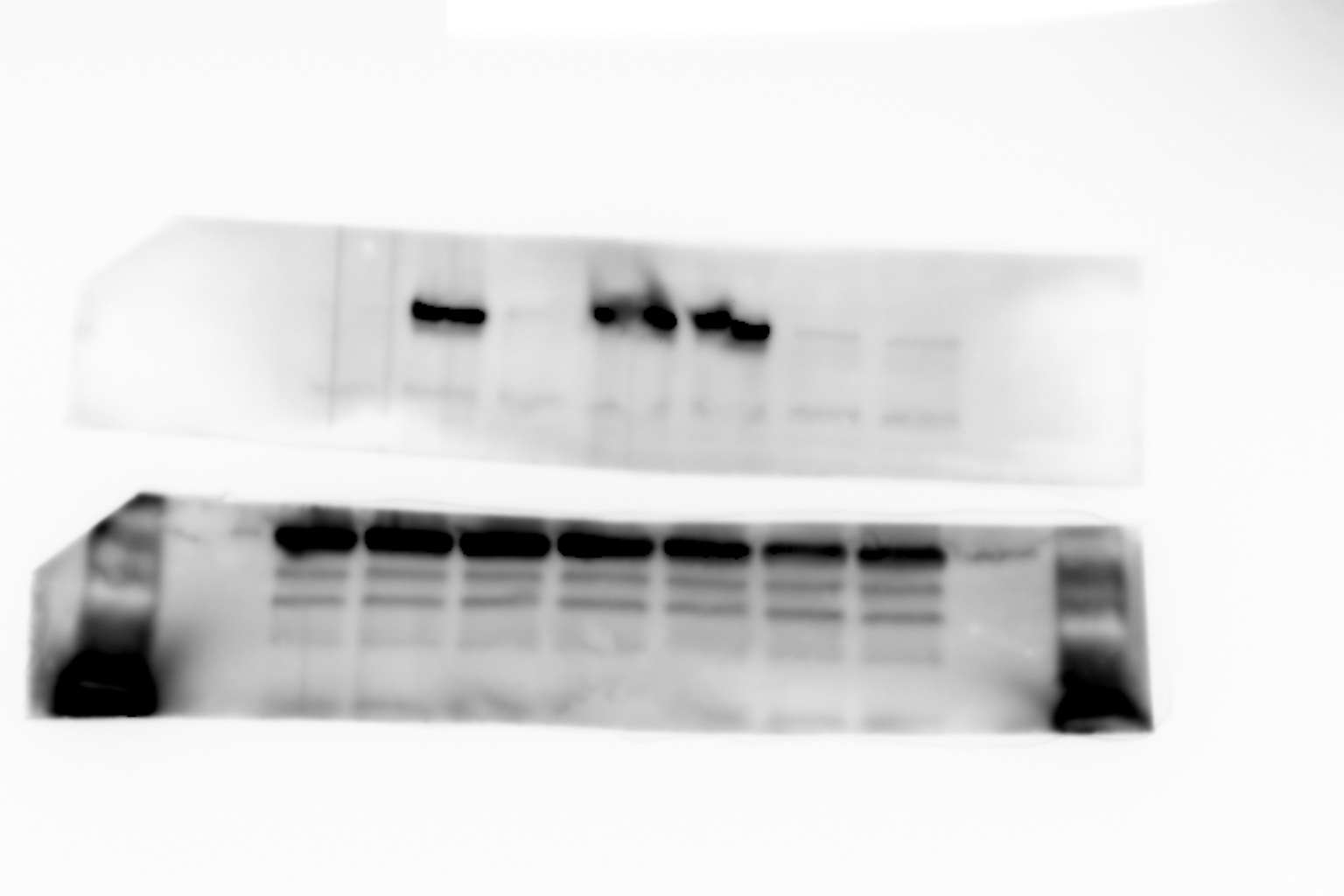

Supplement: S1 File — Original blot images underlying the results of Fig 5B were provided as a zip file. (ZIP) [file pone.0242076.s003.zip › pSTAT GAPDH.jpg]

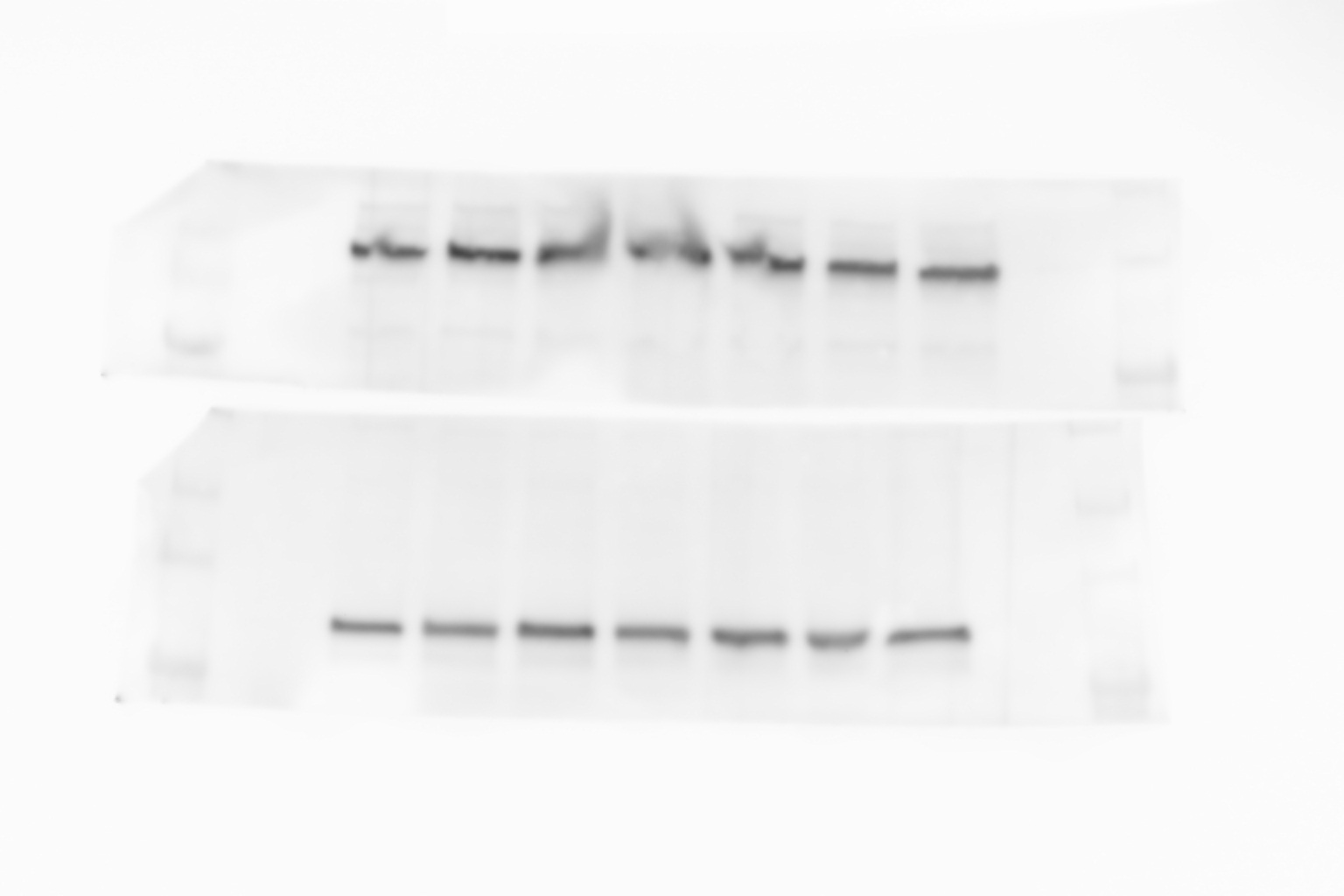

Supplement: S1 File — Original blot images underlying the results of Fig 5B were provided as a zip file. (ZIP) [file pone.0242076.s003.zip › STAT3 NFkB.jpg]
